# Capping the Pulp

**Published:** 1922-08

**Authors:** R. Ottolengui


### CAPPING THE PULP

BY R. OTTOLENGUI, D.D.S.

As final success in pulp capping will depend very largely upon the preliminary treatment, let us consider the technic here expounded. Dr. Norman tells us that he uses an antiseptic; that he dehydrates the dentin to permit a deep penetration. This is exceedingly sound advice, and is emphasized here in order to point out the error of using resins or similar substances. I believe there are pulp capping materials on the market, which take the form of a resin in which has been mixed some antiseptic. Let us study this a moment.

When our much beloved Callahan first introduced his rosin technic for root canal treatment he said that he did not claim that rosin would act as a germicide, but he thought that by surrounding bacteria with an adherent substance of that character he "would greatly increase the high cost of living," for the resident bacteria.

Does not the same hold true of any antiseptic inclosed within the rosin? How can it escape to attack the bacteria? As to the action of the rosin itself, of course as far as it seeps into the decalcified dentin it may disturb the happy dreams of the bacteria within that zone. But what of the bacteria beyond the penetration of the rosin? Will not the rosin actually serve as a barrier between them and any antiseptic? In what direction then will they be forced to travel in search for pabulum? Whither indeed except towards and into the pulp itself? What wonder, then, if that pulp should resent the intrusion, swell up, as it were with rage, thus causing first pain, then strangulation, then death, then abscess, then—Oh! well, you know the list of awful diseases that follow after the tooth abscess.

Probably the best way of comprehending a pulp capping method is first to consider the conditions present and the requirements which the capping should possess.

And here, right or wrong, and until we hear again from Dr. Norman, I must take issue with him where he advises the removal of all decalcified dentin, even though that would uncover the pulp itself. What is decalcified dentin? This is an important query. In the first place, then, as its very name implies, it is dentin. It is dentin altered from its normal structure, but it is dentin in its normal position, so far as the pulp is concerned. The odontoblastic layer of the pulp lies in normal contact with this decalcified dentin, supposing that the decalcification has penetrated thus deeply. How then can any cap fashioned by man, be so accurately fitted over the pulp, as this same decalcified dentin?

But you interrupt me to remark, "Decalcified dentin is filled with bacteria?" Quite so; to some distance at least. But what does that imply? Not the removal of the decalcified dentin, but rather the removal, or at least the destruction, of the bacteria. Can this be done?

Somewhere, at some time (in the *Dental Cosmos*, I think, about 1893 or 1894, if any member wishes to look it up), Prof. Miller, recounting his experiments in relation to caries, made this statement that oil of cloves has the power to destroy bacterial life in decalcified dentin, while at the same time it is the best hardening medium for dentin that could be used in the mouth. I am not sure that he did not say "in the laboratory" as well. What agent can better serve our purpose?

Just consider this for a moment. We have decalcified dentin, leatherly in structure, lying in close and indeed in normal relation with the pulp. We are told that oil of cloves will destroy the bacteria within this mass, and at the same time harden it. Can we do better?

How then may we utilize these qualities? Evidently by placing oil of cloves in contact with the decalcified dentin; by dehydrating that dentin so that the oil of cloves may easily find entrance. Dr. Norman claims that he does this (with whatever antiseptic it is that he uses) in from ten to forty-five minutes. Personally I prefer a week, or even two weeks. But let me pause here to ask, "Do you realize now the folly of gumming up the parts with rosin?"

The preliminary technique for pulp capping then is to remove all decalcified dentin from the walls of the cavity, and as much more as possible, without exposing the pulp. I say as much more as possible, because the less decalcified dentin there is left in the less bacteria there are to kill. Immediately over the decalcified dentin which is permitted to remain, place a pledget of cotton well saturated with pure oil of cloves. Cover this with one of the good temporary cements, placed in the cavity without causing pressure.

Prior to placing the oil of cloves, however, the dehydration spoken of by Dr. Norman should be thoroughly well done so as to permit a ready and deep penetration by the oil of cloves. The warm air blast however is perhaps preferable to alcohol which is a coagulant and might set up a barrier to ingress of the oil of cloves.

The treatment in place, the patient should be dismissed for a week, unless pain should supervene. Sometimes the pulp, though not fully exposed, may be already so badly infected that it will die, in which case pain will result and the patient should report promptly.

Let us suppose that the patient returns after a week, or two weeks in doubtful cases, with no report of pain. The tooth is carefully tested for vitality, and if the pulp be still alive, the operator may proceed with the actual pulp capping.

Once more, before proceeding we should comprehend the necessities of the case. We have used oil of cloves hoping that it would sterilize and at the same time harden the decalcified dentin. But the sterilization may not be complete. One or two bacteria may have escaped.

A goodly number of years ago Dr. Edward Kirk gave me a small bottle of antiseptic pellets saying: "Try that in your root canal work!" "What will it accomplish?" I asked. "Why it will kill the last bacteria, and never forget, that is the one you want to kill. He is not only a long-lived, hardy son-of-a-gun but he sure is prolific when he starts prolificking."

Along similar lines then be it remembered that one or two, yes, or three or four bacteria may still be alive in that decalcified dentin. What is indicated? More oil of cloves, of course.

Next, as to the hardening, Prof. Miller told us that oil of cloves will harden decalcified dentin, but he did not claim that it would become as hard as normal dentin. Hence it is still, in a sense resilient. What of it? Why that will permit stresses to be transmitted to the pulp, and trauma will destroy the pulp in time.

It is evident then that what we need is, first, a prolonged treatment with oil of cloves, and second, a protection over the decalcified dentin that will resist pressure. This is accomplished as follows:

Oil of cloves is mixed with the powder of our oxyphosphate cement into a butter-like consistence. A metallic cap, preferably of platinum to act as a cover. This cap should be made concave, and should be as large as the cavity will admit, so that its edges rest on sound dentin. This consequently forms a bridge over the softened dentin and thus protects the pulp from pressure. For ease of application a tiny point should be cut along the edge of the metallic cap, and this should be turned up at right angles, thus affording a tiny handle, as it were, by which the cap can be taken in the tweezers and carried into position.

The metal cap, held thus in the tweezers is everted and the concaved surface filled with the butter-like paste made of the oil of cloves and powder. It is then placed over the decalcified dentin "butter side down," and tamped to place gently with a small burnisher.

When properly seated, oxy-phosphate cement, mixed to a creamy consistence is flowed over it, care being observed not to dislodge the metal cap, and enough cement being used to form a good protection to the metallic and medicated cap.

By this method we keep oil of cloves in contact with the softened dentin for a maximum of time, and the pulp itself is protected from pressure strains.

It is good practice, if there is time, to continue at this same sitting, while the form and depth of the cavity is fresh in the mind, and after permitting the cement to harden sufficiently, prepare the upper cavity for an inlay, or whatever filling is to be used. By this means the cavity may be filled a month or two later, by simply removing the base plate gutta percha which should have been placed in the cavity.

There is a variation of this procedure which is good practice when the case is considered to be extra hazardous. Place in the bottom of the cavity a considerable quantity of the oil of cloves mixture. Then cut a piece of base plate gutta percha of the same size as the metal cap would have been. Mix oxy-phosphate a little stiff, but not too stiff. Place a generous layer of this on one side only of the piece of gutta percha. Place the gutta percha in the cavity, clean side down, and then spread the cement gently till it touches all the side walls. When hard, this will seal the capping in place, while the gutta percha will prevent the upper cement from mixing with the oil of cloves paste.

This gutta percha cap of course will not afford the protection against pressure that could be gained with the metal cap. Protection against pressure therefore must be gained in another manner. This may be accomplished when preparing the cavity finally for a gold inlay. Ledges may be made in the side walls, or else notches may be cut in the occlusal surface so that the inlay does not actually rest on the cavity floor.

Such an inlay, while in the wax should be specially treated. Immediately over the region of near approach to the pulp, a cavity or box should be cut in the cavo-surface of the inlay. When reproduced in gold this should be deepened until only a very thin layer of gold serves as the top of the box-like cavity. Before setting this inlay, this box should be filled with a temporary stopping, to prevent the ingress of cement.

The object of the various stages of this technique is to prepare against the possibility of pulp death. Should the pulp die after the inlay has been cemented to place, the patient is apt to return with the tooth very sore to the touch. In such event a sharp, spear shaped drill will quickly penetrate the thin gold covering to the cavity which was made on the underside. At this point, if you ever have the experience, you will appreciate finding your drill meet soft temporary stopping rather than hard cement. Further down you will likewise be glad to find the gutta percha cap rather than one of resistant metal.

In actual practice I have only resorted to this method some half dozen times, and only once did the pulp die. But the experience in that instance was sufficiently gratifying to cause me to highly recommend the procedure in very doubtful cases. Indeed the method permits one to "take a chance" that he would not otherwise dare to hazard. In my one case of pulp death, though the tooth was very painful, I readily entered through the gold inlay, and through the underlying materials so that the pulp chamber was quickly opened and relief experienced. Moreover, at a subsequent sitting the cavity through the gold inlay was enlarged, the subsequent sitting the cavity through the gold inlay was enlarged, the root canals cleansed and filled, and the hole in the original large inlay repaired by a small gold inlay, thus avoiding the removal of the first inlay.

As a warning against the indiscriminate and unwarranted use of this pulp capping method I would say that the younger the patient the greater the chances of success. In older patients, or in pulps already infected, disorganized or partly calcified, of course the treatment would often be a failure.—*Dental Items of Interest* in "Around the Table," May 1922.

